# Prognostic and predictive molecular biomarkers in colorectal cancer

**DOI:** 10.3389/fonc.2025.1532924

**Published:** 2025-04-16

**Authors:** Jianzhi Zhang, Hao Zhu, Wentao Liu, Ji Miao, Yonghuan Mao, Qiang Li

**Affiliations:** ^1^ Department of General Surgery, Nanjing Drum Tower Hospital, Affiliated Hospital of Medical School, Nanjing University, Nanjing, China; ^2^ Department of General Surgery, Nanjing Drum Tower Hospital Clinical College of Nanjing Medical University, Nanjing, China; ^3^ Department of General Surgery, Affiliated Drum Tower Hospital, Nanjing University of Chinese Medicine, Nanjing, China; ^4^ Department of General Surgery, Affiliated Drum Tower Hospital, JiangSu University, Nanjing, China

**Keywords:** colorectal cancer, biomarkers, circulating tumor DNA, non-coding RNA, POLE/POLD1, RET

## Abstract

Precision medicine has brought revolutionary changes to the diagnosis and treatment of cancer patients, and is currently a hot and challenging research topic. Currently, the treatment regimens for most colorectal cancer (CRC) patients are mainly determined by several biomakers, including Microsatellite Instability (MSI), RAS, and BRAF. However, the roles of promising biomarkers such as HER-2, consensus molecular subtypes (CMS), and circulating tumor DNA (ctDNA) in CRC are not yet fully clear. Therefore, it is urgent to explore the potential of these emerging biomarkers in the diagnosis and treatment of CRC patients. In this paper, we discuss recent advances in CRC biomarkers, especially clinical data, and focus on the roles of biomarkers in prognosis, prediction, treatment strategies, and the intrinsic connections with clinical pathological features, hoping to promote better precision medicine for colorectal cancer.

## 1 Introduction

Colorectal cancer (CRC) ranks third in global incidence and second in mortality, its epidemiological trajectory showing an alarming upward trend worldwide (1). Although existing screening strategies have moderately reduced mortality rates, the majority of patients are still diagnosed at advanced stages, underscoring an urgent need for transformative approaches in CRC management. The quest for biomarkers with enhanced specificity and clinical utility has therefore become a critical frontier in oncology research by influencing three key areas: early detection accuracy, dynamic monitoring capabilities, and precision therapeutics development. The biomarker revolution in CRC has entered a pivotal phase. Traditional molecular markers such as BRAF and KRAS mutations are widely used in the diagnosis and treatment of colorectal cancer patients (2). However, compared to the revolutionary changes brought by immunotherapy to other cancers (3), the benefits of CRC patients from these biomarker-based treatments are limited, with only 3.8% of mCRC patients with MSI subtypes benefiting from corresponding treatments—a therapeutic gap that highlights the imperative for next-generation biomarker discovery (4). In recent years, with the development of next-generation sequencing, bioinformatics analysis, liquid biopsy, and other technologies, research on biomarkers has entered a new stage: For instance, circulating tumor DNA (ctDNA) has transcended the limitations of tissue biopsies through real-time genomic monitoring, playing a pivotal role in early detection and recurrence monitoring of colorectal cancer patients. Meanwhile, non-coding RNAs (ncRNAs) have unveiled a previously hidden regulatory cosmos, fundamentally reshaping our understanding of CRC pathogenesis. Additionally, RET fusion genes have emerged as targetable oncogenic drivers, demonstrating significant associations with sensitivity to targeted therapies.

n this comprehensive review, we systematically summarize the most recent advancements in biomarker research and critically evaluate their potential for translation into tangible clinical benefits. Our analysis provides an integrative framework that effectively bridges the gap between molecular discovery and clinical implementation, offering valuable insights for both researchers and clinicians in the field of precision medicine.

## 2 Her-2

HER2 is a protein tyrosine kinase receptor encoded on chromosome 17q12, also known as EGFR-2/ErbB-2/CD340. It belongs to the epidermal growth factor receptor (ERBB) family. It has been reported that approximately 7% of CRC patients exhibit alterations of HER2, especially in tumors with wild-type RAS and BRAF. However, the role and impact of HER2 in advanced CRC have not been fully explicit.

### 2.1 The correlation with pathological features

**Figure 1:**
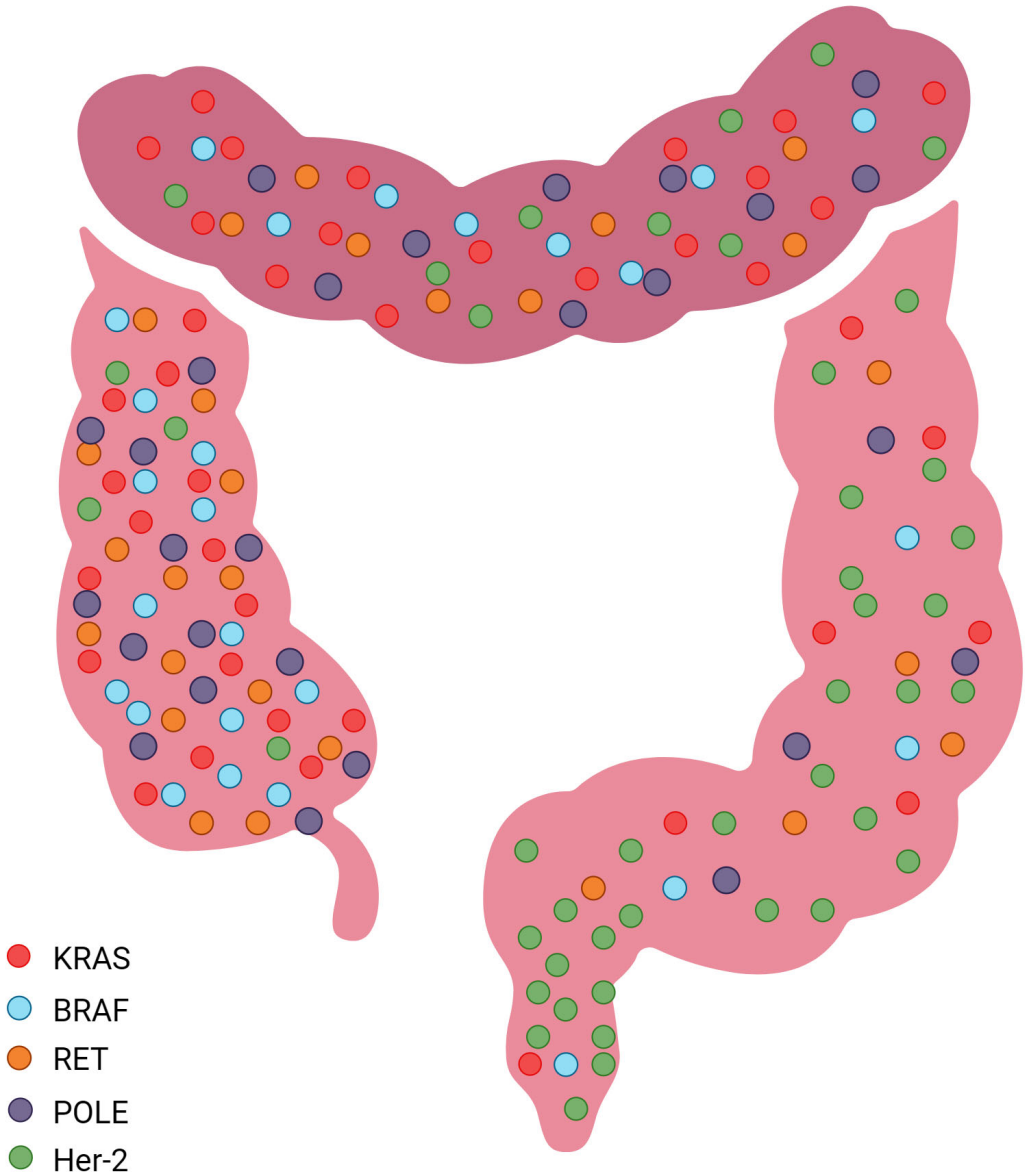
Common sites of molecular markers in the Colorectum.

Tumors overexpressing HER-2 are more commonly found in the left colon or rectum, with an increasing incidence from the right colon to the left colon and then to the rectum (5) (**Figure 1**). The overexpression of HER-2 is significantly associated with higher tumor mutational burden (TMB), higher AJCC staging, and lymph node metastasis (6). Of course, HER-2 also has intrinsic correlations with other biomarkers. For example, point mutations in HER2 are positively correlated with MSI-H tumors, but MSI-H was not found in cases of HER-2 amplification (7). There is also evidence suggesting that the typical molecular subtype (CMS2) is enriched in HER-2 positive tumors, accompanied by changes in epithelial differentiation, WNT, and MYC signaling.

### 2.2 Prognostic value

Currently, the prognostic significance of HER-2 in advanced CRC remains controversial. Compared to HER-2 wild-type tumors, HER-2 amplification in CRC is associated with increased invasiveness and poorer prognosis (6). A retrospective analysis of the PETACC-8 trial (n = 1795) found that HER-2 amplification and mutation were associated with shorter disease-free survival (DFS) (95% confidence interval [CI] 1.02-2.36, P = 0.04) and worse overall survival (OS) (95% CI 0.99-2.5, P = 0.05) Even after adjusting for other prognostic factors such as RAS mutations, grading, tumor location, pT and pN status, bowel obstruction or perforation, lymphatic or venous invasion, the prognostic impact still persisted (8). A study in 2021 supported this view: analysis of 370 mCRC patients found that HER2-positive patients had significantly worse OS compared to patients with low HER-2 expression, indicating potential prognostic value of HER-2 expression in mCRC (9). In contrast, data from the German Rectal Cancer Study Group showed that in 264 rectal cancer patients, HER-2 positivity was associated with better disease-free survival (DFS) and cancer-specific survival (10). However, in a meta-analysis, Richman SD did not find a statistically significant association between HER-2 expression and OS (11). These discrepancies may stem from multiple factors: 1.Anatomical heterogeneity – HER-2’s prognostic impact appears context-dependent, with left-sided colon cancers showing different patterns from rectal tumors; 2.Molecular subtype variations – CMS2-enriched HER-2+ tumors may exhibit distinct biological behaviors; 3.Technical variability in HER-2 assessment methods across studies. The low HER-2 alteration frequency in CRC further complicates conclusive interpretation. This context-dependent prognostic role necessitates standardized molecular subtyping in future studies.

### 2.3 Predictive value

HER-2 alterations have been established as predictive biomarkers of resistance to anti-EGFR therapies, mediating therapeutic escape through two distinct mechanisms: HER-2 gene amplification or heregulin (HRG)-induced HER-3 receptor activation. These pathways converge to constitutively activate downstream ERK1/2 signaling, thereby sustaining oncogenic survival cascades that drive anti-EGFR resistance. However, emerging evidence suggests context-dependent predictive utility: 1. In wild-type KRAS/NRAS/BRAF/PI3KCA populations, HER-2 amplification strongly correlates with shorter response duration to EGFR inhibitors (12, 13). 2.we found that colorectal cancer patients with HER-2 amplification have a shorter duration of response to EGFR monoclonal antibody therapy and worse prognosis compared to those with wild-type RAS/BRAF, approaching even those with RAS or BRAF mutations 3. Recent antibody-drug conjugate (ADC) trials demonstrate HER-2’s positive predictive value for targeted therapies like T-DXd (14). This duality – serving as both resistance marker and therapeutic target – underscores the need for dynamic biomarker assessment throughout treatment courses.

### 2.4 Treatment strategies

Current therapeutic strategies targeting HER-2 include monoclonal antibodies, antibody-drug conjugates (ADCs), and tyrosine kinase inhibitors (TKIs). Although some clinical trial results appear promising, none of these therapies have yet received regulatory approval for metastatic colorectal cancer (mCRC). As previously discussed, HER-2 amplification/mutations mediate resistance to anti-EGFR antibodies, while combination therapy with anti-HER2 and anti-EGFR agents demonstrates synergistic growth inhibition (13). Clinical investigations of pertuzumab-trastuzumab combination therapy in pretreated HER-2-amplified mCRC reveal significant clinical benefit in RAS wild-type HER-2-positive patients, whereas those with RAS mutations show limited therapeutic response (15). Notably, recent progress in TKI development includes pyrotinib—an irreversible dual HER-2/EGFR inhibitor—demonstrating potent antitumor activity when combined with trastuzumab (16). Among ADCs, trastuzumab emtansine (T-DM1) and trastuzumab deruxtecan (T-DXd) have undergone clinical evaluation. Phase II studies of pertuzumab-T-DM1 combination therapy in RAS/RAF wild-type ERBB2-positive mCRC patients demonstrate encouraging efficacy profiles (17).

## 3 BRAF

BRAF is a key serine/threonine protein kinase in the MAPK pathway, including V600 mutations (Class I) and non-V600 mutations. Among them, the Class I BRAF V600E mutation is the most common, accounting for approximately 95%, and exhibits kinase activity 700 times higher than normal BRAF (18). Patients with this type of mutation generally have a poorer prognosis, are typically located in the right colon, more common in females and elderly patients, and are associated with mucinous adenocarcinoma and poorer tumor differentiation (19). At the molecular level, the co-occurrence rate of BRAFV600E mutation and MSI is relatively high, with approximately 52% of MSI tumors having BRAF mutations, while 55% of BRAF mutation tumors exhibit MSI (20).

### 3.1 Prognostic value

Compared to BRAF wild-type CRC patients, patients with BRAF mutations generally have a poorer survival rate. MSI-H tumors carrying BRAF mutations exhibit better OS and lower invasiveness compared to Microsatellite Stability (MSS) tumors with BRAF mutations, suggesting that MSI-H tumors may help mitigate the adverse prognostic impact of BRAF mutations. However, the reported results regarding the prognostic differences among BRAF subtypes are mixed. Patients with BRAF Class I and II mutations generally have a poorer prognosis compared to Class III mutations. On the other hand, another study found that patients with Class II and III mutations seemed to have better survival outcomes (18, 21). Given the dismal prognosis of BRAF V600E-mutant metastatic CRC (mCRC), identifying early efficacy biomarkers post-first-line chemotherapy is critical. Early tumor shrinkage (ETS) and depth of response (DpR) serve as quantifiable metrics for initial treatment assessment. At the 2024 Japanese Society of Medical Oncology (JSMO) conference, clinical data validated these parameters as prognostic surrogates in BRAF V600E-mutant mCRC patients undergoing first-line chemotherapy (22).

### 3.2 Treatment strategies

#### 3.2.1 Chemotherapy combined with anti-VEGFR antibodies

The first-line treatment choice for mCRC patients is dual (FOLFIRI/FOLFOX/CAPOX) or triple (FOLFOXIRI) chemotherapy combined with bevacizumab (anti-VEGFR antibody) (23). Subsequent studies have shown that triple chemotherapy increases toxicity compared to dual chemotherapy regimens, with almost no difference in actual efficacy (24). However, patients with right-sided CRC do benefit from triple chemotherapy regimens (25). Currently, the ESMO clinical practice guidelines recommend dual chemotherapy + bevacizumab for MSS-type BRAF V600E-mutated CRC patients, with triple chemotherapy + bevacizumab being reserved for special circumstances (such as tumors located on the right side).

#### 3.2.2 BRAF inhibitors

Previous studies have indicated that BRAF inhibitors alone do not achieve satisfactory outcomes, as they can lead to feedback activation of EGFR and reactivation of the MAPK pathway. Therefore, the combination of the BRAF inhibitor encorafenib with the anti-EGFR antibody cetuximab is considered the optimal choice for second-line treatment in MSS-type BRAF-mutated mCRC patients (23). Recently, a study added nivolumab to the above regimen and investigated the outcomes of combination therapy. The current results show that combination therapy is more effective and well-tolerated by patients (26).

#### 3.2.3 Immunotherapy

About 70% of BRAF mutation tumors belong to CMS1 type, suggesting that MSI-H mCRC patients with BRAF mutations may benefit from immunotherapy (27). KEYNOTE-177 and CHECKMATE 142 evaluated the efficacy of pembrolizumab and nivolumab monotherapy in BRAF-mutant MSI-H mCRC patients, respectively. The results showed that immunotherapy was more effective than traditional therapy (28, 29). CheckMate-142 also evaluated the efficacy of ipilimumab + nivolumab combination therapy, and the results showed that the combination therapy was more effective than nivolumab alone (30). In addition, when relatlimab was used in combination with PD-1 blockade, it significantly slowed CRC tumor formation in mice (31). Therefore, we speculate that anti-LAG-3 monoclonal antibodies may enhance the efficacy of immune checkpoint inhibitors (ICIs) in CRC patients.

## 4 KRAS

The RAS protein family, classified as small GTPases, comprisesHRAS, NRAS, and KRAS, which are among the most commonly mutated genes in human cancers. In colorectal cancer, KRAS mutations are the most common (43%), followed by NRAS (9%) and HRAS (1%).

Kirsten rat sarcoma viral (KRAS), also known as the P21 gene, is a commonly mutated gene in cancer. In KRAS mutations, 97% (to be verified) involve mutations in the 12th or 13th amino acid residues, with the most common being G12D, G12V, and G13D mutations. KRAS mutations are more common in the right colon and are associated with advanced disease stage, poorly differentiated tumors, distant metastasis, and poorer survival rates. They are also more common in females and younger patients (32). Additionally, KRAS mutations are associated with shorter time to recurrence (TTR), recurrence-free survival (SAR), and OS in patients with non-microsatellite instability (MSI) tumors (33).

### 4.1 Prognostic value

KRAS mutations are closely associated with the occurrence and development of CRC, with studies indicating that patients with KRAS-mutant CRC generally have a worse prognosis compared to those with wild-type KRAS. Prognostic heterogeneity exists across mutation subtypes: One study showed that mutations in codon 12 were significantly correlated with both OS and Disease-free survival (DFS), particularly G12D and G12V mutations (34). Meanwhile, G12C mutations may represent a poorer prognosis, while the prognosis for patients with G12D mutations may fall between wild-type and G12C mutations (35). The prognostic significance of mutations in codon 13 remains controversial (34). A study based on the double-blind, controlled, phase 3 RECURSE trial and using two independent datasets demonstrated that codon-specific KRAS mutations could predict the clinical benefits of patients with mCRC receiving chemotherapy with trifluridine/tipiracil (FTD/TPI): patients with KRASG12 mutations did not benefit significantly from FTD/TPI chemotherapy in terms of OS, whereas KRASG13 mutations represented a poorer prognosis and had better efficacy with FTD/TPI (36). Rare variants (e.g., G12F, G13C) remain understudied, with limited data on their clinical implications.

### 4.2 Predictive value

KRAS mutations are also considered predictive markers for poor response to chemotherapy combined with anti-EGFR treatment: this is because when KRAS mutates, it remains in a constitutively active state by continuously binding to GTP, thereby bypassing the activating effect of EGFR ligands. However, not all KRAS mutations confer resistance to anti-EGFR treatment. Previous studies have shown that colorectal cancer patients with G13D mutations benefit from first-line chemotherapy plus cetuximab, but their progression-free survival (PFS), OS, and response rates(RR) are still lower than those of patients with wild-type KRAS tumors (37). Currently, the combination of bevacizumab (anti-VEGFR) with chemotherapy is considered the best first-line treatment for patients with KRAS-mutant mCRC (38).

### 4.3 Treatment strategies

#### 4.3.1 Direct inhibition

Currently, directly inhibiting the KRAS gene is highly challenging for several reasons. Firstly, due to the exceptionally high affinity of KRAS for GTP and GDP, developing a competitive small molecule inhibitor is extremely difficult. Secondly, KRAS has a broad range of functions, and inhibiting KRAS may lead to significant toxicity. Moreover, designing a drug that selectively inhibits mutated KRAS without affecting normal KRAS is not straightforward. Presently, two KRASG12C inhibitor: Adagrasib and Sotorasib, have shown significant efficacy in non-small cell lung cancer, but their efficacy in CRC remains limited (39, 40). However, when used in combination with other drugs, such as adagrasib combined with cetuximab (response rate 43%, disease control rate 100%), the efficacy is remarkably improved (41). Similarly, sotorasib in combination with panitumumab also has a beneficial effect on improving patient prognosis (42). Studies have also evaluated the effects of KRASG12C inhibitors combined with anti-PD-L1 therapy and found that the combination leads to a further increase in the number of CD3+ T cells and CD8+ T cells in patients, offering a promising therapeutic approach (43).

#### 4.3.2 Nucleotide exchange inhibitors

Recently, researchers have discovered BAY-293, which is an inhibitor that disrupts the binding of SOS1 protein to KRAS. Meanwhile, BI-3406 is a more effective and selective SOS1 inhibitor that only inhibits SOS1 without affecting SOS2 (44). SHP2 inhibitors, similar to SOS1 inhibitors, can prevent the loading of GTP on RAS. Currently, SHP2 inhibitors are in the early stages of clinical trials, such as rmmc-4630 and TNO155, with TNO155 found to enhance the efficacy against KRASG12C-mutant CRC when used in combination with KRASG12C covalent inhibitors (45). Both types of inhibitors can suppress tumor growth, and experiments targeting these inhibitors are currently underway.

#### 4.3.3 Inhibiting KRAS-related signaling pathways

Inhibiting the KRAS-related signaling pathway is another approach for treating patients with KRAS-mutant CRC. RAF is a direct downstream effector of KRAS, and selective inhibition of RAF may have limited therapeutic efficacy due to feedback loops or RAF dimerization activating MEK (46). Therefore, the therapeutic effect of selective RAF inhibition is limited. Researchers have utilized pan-RAF inhibitors that block RAF dimer-dependent signaling, such as belvarafenib, which has shown promising anti-tumor activity (47). In addition, MEK inhibitors such as Selumetinib, Trametinib, cobimetinib, have demonstrated good efficacy, but MEK inhibitors may have significant toxicity issues that need to be addressed, and simultaneous inhibition of RAF and MEK may be a better treatment option. Furthermore, inhibiting ERK1/2 may overcome the limitations of upstream RAF or MEK inhibitors, and LY3214996 (an ERK1/2 inhibitor) has shown promising anti-tumor activity in preclinical studies and acceptable safety in trials, further supporting its efficacy as monotherapy or in combination therapy (48, 49). Inhibiting the PI3K-AKT-mTOR pathway is another effective approach: the triple combination of PI3K inhibitor alpelisib + encorafenib (a BRAF inhibitor) + cetuximab (anti-EGFR monoclonal antibody) has shown encouraging results in mCRC patients (50). It is worth noting that most mTOR inhibitors have poor efficacy as monotherapy (51).

Inhibiting one pathway can lead to compensatory activation of another pathway, so simultaneously inhibiting MAPK/PI3K is a promising strategy. Given compensatory pathway activation during single-target inhibition, concurrent MAPK/PI3K blockade emerges as a rational strategy. While preclinical models support PI3K/MEK inhibitor synergy against KRAS-mutant tumors (52). However, in recent clinical trials, the tolerability and activity of these inhibitor combinations have not been satisfactory (53).

Other emerging therapeutic approaches, such as targeting tumor metabolism processes, KRAS-targeted siRNA, anti-RAS vaccines, offer hope for inhibiting tumor growth and providing a potential treatment option for KRAS-mutant colorectal cancer. However, their clinical efficacy remains to be further validated.

## 5 MSI

Microsatellite Instability (MSI) refers to a type of repetitive DNA sequences present in the human genome. Due to the high-frequency repeats of these sequences, errors are prone to occur during replication, and cells rely on DNA mismatch repair proteins (MMR) to correct these errors. However, when MMR function is deficient (dMMR), replication errors in microsatellites cannot be corrected, leading to the accumulation of sequence length or composition changes, resulting in microsatellite instability (MSI). Based on the detection of loci, MSI is classified into MSS, MSI-L, and MSI-H. Previous studies have shown that MSI-L has no significant biological differences compared to MSS tumors, so MSI-L is often grouped with MSS in clinical practice.

### 5.1 The correlation with pathological features

dMMR/MSI-H CRCs are more commonly found on the right side, mostly presenting as mucinous adenocarcinomas, and are closely associated with poorly differentiated tumors (54). Researchers have observed an increased occurrence of BRAF mutations in advanced dMMR/MSI-H CRC tumors. In fact, preclinical data suggests that the BRAF V600E mutation can promote the dMMR/MSI-H phenotype by activating the MAPK pathway. Additionally, influenced by the tumor microenvironment (TME), approximately 70% of dMMR/MSI-H CRCs cluster within the CMS1 subtype (55).

### 5.2 Prognostic value

dMMR/MSI-H is more commonly observed in the early stages of tumors, which may be due to the high tumor mutational burden (TMB) in early-stage colorectal cancer generating abundant neoantigens, activating CD8+ T cell infiltration, forming an immunogenic microenvironment that inhibits tumor progression. However, when tumors metastasize, dMMR/MSI-H becomes a negative prognostic factor, which may be related to the confounding effect of BRAF mutations: in advanced MSI-H CRC, the BRAF V600E mutation rate reaches up to 20%, suggesting that this prognostic difference may be driven by BRAF mutations rather than MSI itself.

### 5.3 Treatment strategies

Although colorectal cancer patients with MSI-H generally exhibit favorable responses to ICIs such as anti-PD-1, PD-L1, or CTLA-4 antibodies (56), emerging evidence has revealed critical modifying factors: 1. Spatial heterogeneity: Ascites-associated peritoneal metastases significantly diminish ICI efficacy, likely resulting from the interplay between malignant ascites and an immunosuppressive tumor microenvironment (57); 2. Microbiome interference: The use of broad-spectrum antibiotics (ATBs) negatively impacts ICI therapeutic efficacy, potentially due to their disruption of the gut microbiota and consequent adverse effects on immune function (58), Conversely, leveraging gut microbiota to enhance immunotherapy shows promise; for instance, Fusobacterium nucleatum has been shown to potentiate the anti-tumor effects of PD-L1 blockade in colorectal cancer (59). These findings challenge the paradigm of MSI-H as a standalone predictive biomarker and underscore the necessity of adopting a composite biomarker strategy.

## 6 MSS

Microsatellite Stable (MSS) refers to a phenotype in colorectal cancer (CRC) where microsatellite sequences maintain stable length and composition during replication, with its pathogenesis being closely associated with proficient mismatch repair (pMMR) functionality. Unlike MSI-H/dMMR-type CRC, MSS/pMMR tumors exhibit low tumor mutational burden (TMB) and reduced immune cell infiltration within the tumor microenvironment (TME), typically manifesting as a ‘cold tumor’ phenotype. These characteristics result in poor response to immune checkpoint inhibitor monotherapy (60).

### 6.1 The correlation with pathological features

MSS-type CRC accounts for about 85% to 90% of all colorectal cancers, mostly found in the left half of the colon and rectum, and the histology is predominantly adenocarcinoma with a high degree of differentiation. Notably, the immune microenvironment of MSS-type CRC is dominated by suppressive immune cells (e.g., T regulatory cells, M2-type macrophages), and the expression level of PD-L1 is generally low, leading to active immune escape mechanisms (61).

### 6.2 Prognostic value

The prognosis of MSS-type CRC is strongly correlated with tumor stage and tumor microenvironment (TME) characteristics. While early-stage MSS patients demonstrate comparable survival outcomes to MSI-H cases, advanced-stage MSS patients exhibit significantly poorer survival rates than their MSI-H counterparts, potentially attributable to chemotherapy resistance and immunosuppressive TME. Recent studies have identified tumor-infiltrating lymphocyte (TIL) density as a critical prognostic biomarker in MSS CRC: Compared with MSI-TIL-H subtypes, MSS-TIL-H patients maintain microsatellite stability yet paradoxically demonstrate superior survival advantages. Specifically, MSS-TIL-H patients show significantly improved overall survival (HR = 0.53) and disease-free survival (HR = 0.52) compared to MSS-TIL-L subgroups (62). This finding highlights that elevated TIL infiltration confers substantial survival benefits even within the MSS context.

### 6.3 Treatment strategies

Modulating the immune microenvironment of MSS colorectal cancer to convert ‘cold tumors’ into ‘hot tumors’ has become particularly crucial in refractory MSS-type CRC, especially for advanced metastatic patients. Current clinical strategies to enhance immunotherapy efficacy in CRC primarily focus on two approaches: 1. Combination Therapies: ①Immune-targeted combinations: Emerging evidence suggests fruquintinib (a VEGFR inhibitor) combined with PD-1 inhibitors represents a promising therapeutic option for refractory MSS metastatic CRC (mCRC), demonstrating tolerable toxicity. Notably, incorporation of local therapies in patients with liver metastases may significantly extend overall survival (OS) (63). ②Dual immunotherapy: Early-phase trials indicate that botensilimab (BOT, a multifunctional CTLA-4 inhibitor) combined with balstilimab (BAL, a PD-1 blocker) achieves durable responses and prolonged OS across all subgroups while maintaining a favorable safety profile (64); 2. Biomarker-driven Strategies:Identification of predictive molecular biomarkers for immunotherapy response in MSS CRC, including previously discussed tumor-infiltrating lymphocytes (TILs), POLE/POLD mutations, and tumor mutational burden (TMB). These biomarkers may enable patient stratification to optimize therapeutic outcomes.

## 7 CMS

The Consensus Molecular Subtypes (CMS) is a widely used molecular classification method in colorectal cancer, which divides tumors into four subgroups (CMS1-4) based on mRNA gene expression patterns:CMS1 (MSI immune subtype): Associated with MSI, dDNA mismatch repair, BRAF V600E mutation, hypermutation (Abnormally accelerated gene mutation rates, often in immune cells to rapidly generate antibody variants), CIMP, and primarily observed in females. These tumors have higher histopathological grades and poorer survival rates after recurrence.CMS2 (Canonical subtype): Characterized by chromosomal instability, immune desert (Tumor regions with minimal immune cell presence), TP53 mutation, and upregulation of the EGFR pathway. Tumors in this subtype are typically located on the left side.CMS3 (Metabolic subtype): Typically characterized by abnormalities in metabolic pathways, KRAS mutation, and lower levels of CIMP and CIN.CMS4 (Mesenchymal subtype): Characterized by upregulation of EMT and SCNA, chromosomal instability, and constitutive activation of VEGFR and TGF-β pathways. CMS4 tumors are primarily associated with advanced stages (III and IV).CMS1 and CMS4 are associated with immune infiltration and considered “hot” tumors, while CMS2 and CMS3 are the opposite, characterized as “cold” tumors in terms of immune response.

### 7.1 Prognostic value

#### 7.1.1 Local

The PETACC-3 study found that compared to other subtypes, CMS4 subtype has a significantly worse prognosis, and the same conclusion was drawn after adjusting for KRAS, BRAF, and MSI status (65). This may be related to the higher expression levels of monocytes, lymphocytes, and inflammatory and immune suppressive characteristic factors in the CMS4 subtype.

#### 7.1.2 Metastasis

If the tumor metastasizes distantly, CMS1 exhibits the worst prognosis in terms of OS and PFS compared to other subtypes, consistent with its higher BRAF V600E mutation rate and the negative prognostic impact of MSI (66). CMS2 generally has a better prognosis, while the prognosis of CMS3 and CMS4 falls between the two (66).

### 7.2 Predictive value

#### 7.2.1 Chemotherapy

Research indicates that CRC patients with the CMS3 subtype only benefit from chemotherapy in stage III (p = 0.001), while patients with the CMS2 subtype (stage II and III) show improved survival rates after receiving adjuvant chemotherapy (p = 0.02 and p < 0.001) (67). However, CMS1/CMS4 subtypes show no survival advantage from adjuvant chemotherapy (68).

#### 7.2.2 Targeted therapy

The FIRE-3 trial included 514 mCRC patients who were randomly assigned to receive first-line treatment with FOLFIRI plus bevacizumab or cetuximab. The results revealed a better prognosis for CMS2 (29 months), while CMS4 (24.8 months) and CMS3 (18.6 months) had intermediate prognoses. CMS1 subgroup showed the shortest survival, with only 15.9 months (69). Consistent conclusions were drawn by the CALGB/SWOG 80405 trial, which also found that compared to FOLFIRI plus cetuximab, bevacizumab treatment was more effective for CMS1 tumors. This is consistent with their characteristics: CMS1 is more commonly associated with BRAF mutations or RAS mutations, which lead to resistance to anti-EGFR therapy, making patients more responsive to bevacizumab. Conversely, a completely opposite scenario was observed in the CMS2 subtype, which may be because CMS2 is more common in left-sided tumors, where anti-EGFR drugs are more effective (70).

CMS3 tumors can also benefit from treatment with bevacizumab plus capecitabine, with significant improvements in both PFS and OS (71). In CMS4, patients receiving bevacizumab plus FOLFIRI often experience better PFS and OS compared to chemotherapy alone. However, when bevacizumab is replaced with capecitabine, patients show better prognosis (71).

A recent study from Panama included 296 RAS wild-type mCRC patients and evaluated the efficacy of panitumumab (Pmab) plus fluorouracil/leucovorin (FU/FA) in various CMS types. The results showed beneficial outcomes with Pmab + FU/FA in CMS2/4 tumors, while no efficacy was observed in CMS1/3 tumors (72).

#### 7.2.3 Immune therapy

Given the characteristics of CMS1 subtype, (ICIs) may be an effective approach for treating patients with this subtype. A study presented at ASCO GI 2022 demonstrated that combining Nivolumab with standard treatment in CMS1 and CMS3 subtypes could potentially yield clinical benefits (73).

## 8 Circulating tumor DNA

**Figure 2:**
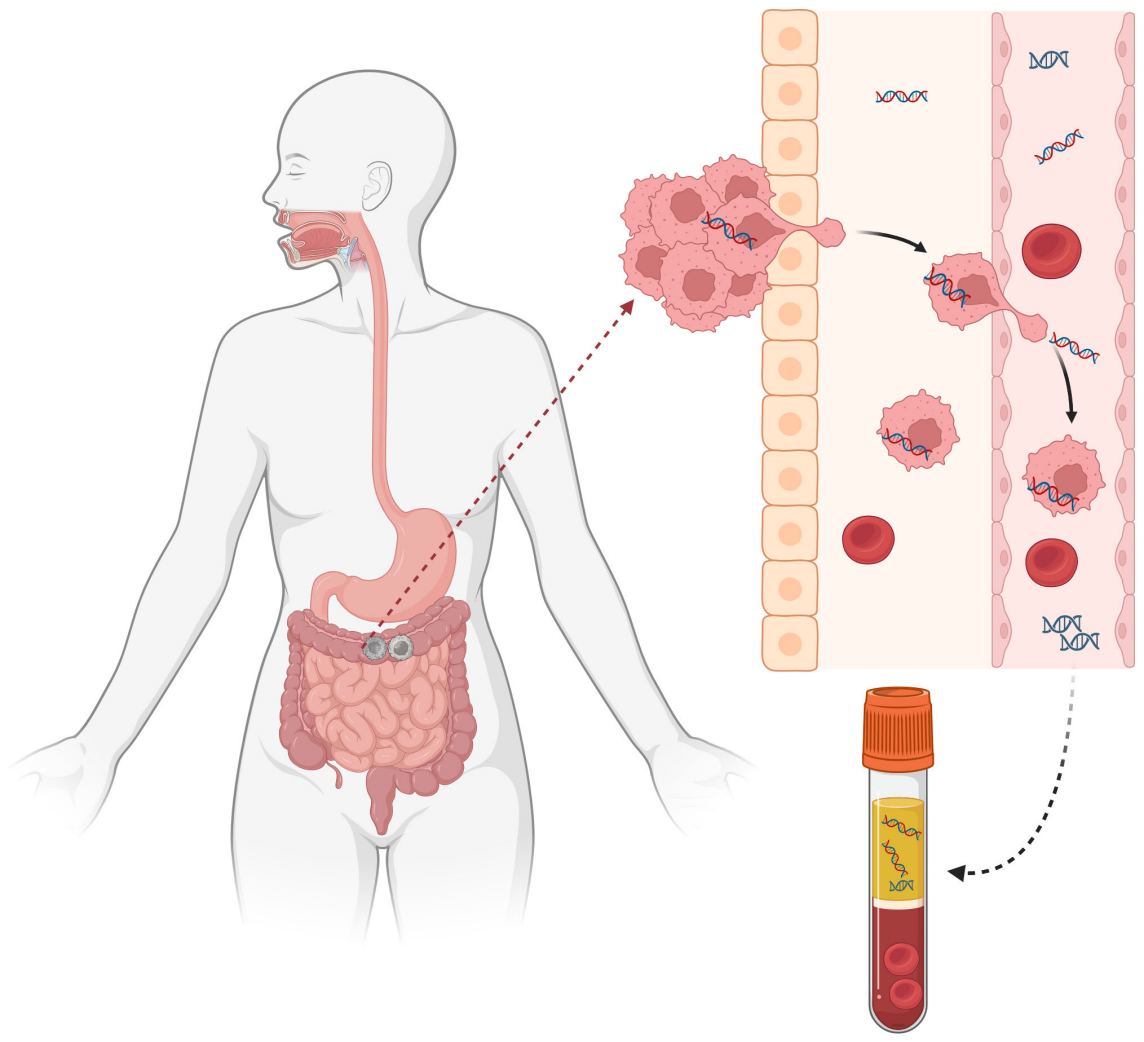
Liquid biopsy.

The small fragments of DNA released into the bloodstream after cell apoptosis or necrosis are called circulating free DNA (cfDNA), among which DNA released by tumor cells is referred to as circulating tumor DNA (ctDNA). Liquid biopsy utilizes peripheral blood extraction for ctDNA analysis, enabling real-time monitoring of tumor evolution. This approach provides three key advantages over conventional biopsies:1. Minimally invasive procedure; 2. Flexible temporal sampling; 3. Simplified specimen storage (**Figure 2**). Currently, ctDNA serves as a powerful biomarker closely associated with patient prognosis and can predict recurrence in CRC patients. Additionally, ctDNA detection can provide relevant molecular profiles (such as RAS/RAF/HER), replacing tissue sequencing to guide subsequent treatment.

### 8.1 Prognostic value

In 2019, literature reported on the prognostic role of ctDNA in stage I-III CRC patients. The study included 125 patients and collected a total of 829 plasma samples. The findings revealed that patients positive for ctDNA, whether postoperative or post-ACT (adjuvant chemotherapy), were associated with a high risk of recurrence. Moreover, patients negative for ctDNA had a significantly better prognosis compared to ctDNA-positive patients, with ctDNA proving to be a more valuable prognostic indicator than radiological parameters (74). Gong Chen and colleagues confirmed this conclusion, demonstrating that even after adjusting for known clinical and pathological risk factors, ctDNA positivity remained the most important independent predictor of disease-free survival in stage II-III colorectal cancer patients (75). It’s worth noting that only postoperative ctDNA minimal residual disease (MRD) can predict the prognosis of postoperative CRC patients, even identifying those at high risk of recurrence, while preoperative ctDNA testing cannot predict patient prognosis (76). Another study, which merged patient data from three studies and had a follow-up period exceeding 5 years, confirmed the prognostic value of ctDNA. It found that ctDNA had higher predictive accuracy for recurrence-free survival (RFS) compared to individual clinical and pathological risk factors. When combined with all clinical variables, ctDNA significantly improved the accuracy of recurrence prediction (77).

### 8.2 Predictive value

ctDNA serves not only as a prognostic marker in colorectal cancer (CRC) but recent research suggests it may also function as a predictive biomarker for treatment response. A study on IDEA-France’s post hoc analysis revealed that irrespective of clinical high-risk factors, stage III CRC patients positive for ctDNA had better outcomes with a 6-month chemotherapy regimen compared to 3 months (78). To evaluate whether a ctDNA-guided approach could reduce adjuvant chemotherapy use without compromising recurrence risk, researchers, led by Jeanne, randomly allocated 441 stage II colon cancer patients in a 2:1 ratio based on ctDNA and clinical-pathological characteristics to guide treatment decisions. The results showed that ctDNA-negative patients maintained high 2-year disease-free survival (DFS) without adjuvant chemotherapy. Additionally, although the proportion of patients receiving adjuvant chemotherapy was lower in the ctDNA-guided group, it did not affect survival, and the efficacy was non-inferior to the standard management (79).

A study based on the phase III PARADIGM trial explored the predictive value of ctDNA negativity for RAS wild-type mCRC patients regarding panitumumab selection (no predefined resistant gene mutations detected). Findings suggest that ctDNA-guided molecular selection (rather than primary tumor location) identifies patients likely to benefit from first-line panitumumab-chemotherapy combinations (80). A recent study found that ctDNA may predict treatment choice for gastrointestinal stromal tumors (GISTs) treated with either ripretinib or sunitinib. Analysis of KIT exon mutations in peripheral blood ctDNA showed better efficacy with sunitinib in patients with KIT exon 11 + 13/14 mutations, while patients with KIT exon 11 + 17/18 mutations had better progression-free survival (PFS) with ripretinib (81).

Furthermore, ctDNA also has the ability to predict the emergence of acquired resistance, being more convenient and sensitive than traditional tumor biopsies. For example, ctDNA exhibits higher sensitivity to acquired RAS mutations, allowing us to exploit this advantage to circumvent acquired resistance to anti-EGFR therapy. The NCT04776655 trial is a prospective randomized phase III study based on ctDNA aimed at evaluating the optimal monoclonal antibody therapy in mCRC patients with RAS/BRAF wild-type and liquid biopsy RAS mutations. In addition to anti-EGFR drugs, ctDNA has been validated to have predictive value in targeted therapies such as anti-HER-2, anti-BRAF/EGFR, and KRASG12C-directed therapies (2, 82, 83). On the other hand, (ICIs), as emerging treatments in recent years, are also closely associated with ctDNA. The ARETHUSA clinical trial treated pMMR, RAS-mutated mCRC patients with temozolomide (TMZ). Analysis of ctDNA showed that TMZ treatment resulted in MMR deficiency, increased TMB, increased sensitivity to immune therapy, and ctDNA can accurately measure blood TMB (bTMB) and predict the efficacy of pembrolizumab, similar to previous Canadian study results. Thus, ctDNA can be considered a marker for assessing TMZ efficacy (84).

## 9 Non-coding RNA

Non-coding RNAs (ncRNAs) comprise >90% of the human transcriptome despite lacking protein-coding capacity. These molecules critically regulate protein biosynthesis, cellular homeostasis, and transcriptional networks. Mounting evidence implicates ncRNAs—particularly microRNAs (miRNAs), long noncoding RNAs (lncRNAs), and circular RNAs (circRNAs)—in colorectal carcinogenesis and progression.

Mechanistically, ncRNAs modulate colorectal cancer phenotypes via STAT3 pathway regulation and epithelial-mesenchymal transition (EMT) modulation. Their dysregulated expression patterns have emerged as multifunctional biomarkers for prognosis prediction, therapy response assessment, and drug resistance targeting in colorectal cancer.

### 9.1 MicroRNA

MicroRNAs (miRNAs) are non-coding, single-stranded short RNA sequences that regulate gene expression and influence biological behaviors such as cell proliferation, differentiation, and apoptosis. Both low and high expression of miRNAs can potentially impact the initiation, progression, and prognosis of tumors. Certain miRNAs have been demonstrated to be associated with the clinical and pathological characteristics of colorectal cancer (CRC) patients. For instance, compared to healthy individuals and benign adenomas, miR-874 is downregulated in CRC patients, and its downregulation is associated with advanced tumor stage, lymph node metastasis, and distant metastasis, serving as an independent prognostic factor for CRC (85). Wang et al., through ROC curve analysis, found that miR-377-3p and miR-381-3p can serve as diagnostic biomarkers for early-stage CRC (86). Additionally, the combination of miRNA and CEA for CRC diagnosis has been shown to improve diagnostic accuracy, such as the combination of miR-150-5p and CEA (87).

The miRNA/STAT3 axis regulates CRC tumors by influencing EMT, thereby affecting patient prognosis. For example, upregulation of miR-34a, miR-200b, miR-27a, and miR-330 can decrease the proliferation and invasion capacity of CRC tumor cells (88, 89). However, miR-22 serves as a crucial regulatory factor; it downregulates MAX to inhibit EMT and consequently suppresses the expression of NLRP3, leading to reduced invasive and metastatic abilities of CRC (90). On the contrary, the high expression of certain miRNAs can promote EMT and thus facilitate the progression and metastasis of CRC (such as miR-645) (91).

Furthermore, miRNAs have good predictive value for chemotherapy resistance in CRC patients. In recent years, it has been discovered that overexpression of HIF‐1α under hypoxia can intervene in patients’ resistance to oxaliplatin by reducing the level of miR‐338‐3p in the blood (92). In 2021, Chinese scholars found that upregulation of miR-208b can target PDCD4, enhancing patients’ resistance to oxaliplatin (93).

### 9.2 Long non-coding RNA

Long non-coding RNAs (lncRNAs) are relatively stable and can participate in tumor progression through various pathways, including competitive inhibition with miRNAs, regulation of tumor cell stemness, influence on RNA-binding proteins, and intervention in cell autophagy. Therefore, they can serve as biomarkers for diagnosis, prognosis, and prediction in CRC.

#### 9.2.1 The correlation with pathological features and prognostic value

Compared to healthy individuals, many CRC patients exhibit significantly elevated levels of serum lncRNAs, which are often associated with poorer prognosis. One representative example is Colorectal Cancer Associated Transcript (CCAT), whose overexpression has been shown to correlate with increased tumor invasiveness and lymph node metastasis. RPPH1 is another lncRNA confirmed to be associated with clinical pathological features, with its high expression in tumor tissues correlating with later stage and poorer prognosis, both of which can serve as diagnostic and prognostic markers. Other lncRNAs found to be associated with TNM staging include GLCC1, which, when combined with TNM staging, can more accurately analyze CRC prognosis.

#### 9.2.2 Predictive value

LncRNAs can promote or inhibit EMT, thereby affecting the occurrence and development of CRC and patient prognosis. Some lncRNAs can induce EMT and promote colorectal cancer invasion and metastasis by regulating miRNAs. For example, lncRNA CASC21 (94) and lncRNA XIST (95) can downregulate their target miRNAs. Therefore, targeting the lncRNA/EMT axis holds promise as a new therapeutic approach for treating CRC patients. Additionally, lncRNAs have predictive value for treatment selection. For instance, MIR100HG, UCA1, CRART16, SLCO4A1AS1, and TTN-AS1, whose high expression can enhance patient sensitivity to cetuximab and panitumumab (96). In 2022, Qiu et al. combined H&E images with deep learning and found significant differences in mRNA, miRNA, and lncRNA between MSI-H and MSI-L/MSS patient groups (97). Also in the same year, a study found that LINC00963 is highly expressed in CRC patients and is associated with increased response to MSI-H and immunotherapy (98). LncRNAs are also associated with genes related to KRAS mutations. Additionally, lncRNAs interact with KRAS-mutant pathways: An Iranian cohort study identified 12 prognosis-linked lncRNAs (including SSTR5-AS1 and RASSF8-AS1) that modulate Rap1/RAS signaling networks (99).

### 9.3 Circular RNA

Circular RNAs (circRNAs) are a type of RNA with a more stable covalently closed-loop structure, which functions include acting as miRNA sponges, interacting with mRNA, regulating transcription, and protein translation. Currently, there are various methods for detecting circRNAs, such as reverse transcription quantitative polymerase chain reaction (RT-qPCR), droplet digital PCR (ddPCR), microarray analysis, RNA sequencing (RNA-seq), Northern blotting, fluorescence in situ hybridization (FISH), NanoString technology, and more. Increasing evidence suggests that circRNAs play a crucial role in the pathological and physiological functions (such as proliferation, migration, etc.) and drug resistance of tumor cells.

#### 9.3.1 Prognostic value

There are significant differences in circRNA expression between normal tissues and colorectal cancer tissues. For example, circHERC4 is upregulated in colorectal cancer tissues and positively correlated with advanced tumor stage (100). Conversely, circPLCE1 is downregulated in colorectal cancer tissues and associated with poorer prognosis and advanced clinical stage (101). In a study involving 1430 colorectal cancer patients, it was found that the differential expression of circRNAs in cancer tissues is often associated with tumor size, differentiation, TNM staging, invasiveness, lymph node, and distant metastasis. Patients with low circRNA expression tend to have better prognosis and longer survival, while the opposite is true for those with high expression (102). These findings confirm the significant potential of circRNAs as diagnostic and prognostic markers. Additionally, some circRNAs have been found to effectively predict the resistance to colorectal cancer treatment, such as circ_0000236 (103) and circ-ZEB1 (104), which are associated with chemotherapy resistance in CRC. circLHFPL2 and circIFNGR2 are significantly associated with resistance to cetuximab and MEK inhibitors (105, 106).

#### 9.3.2 Predictive value

CircRNAs associated with tumor cells may become new therapeutic targets. Animal experiments have shown that short hairpin RNAs (shRNAs) targeting circMETTL3 can inhibit tumor growth and metastasis (107). Many other animal experiments have confirmed this viewpoint, indicating that targeting cancer-related shRNAs or small interfering RNAs (siRNAs) may inhibit the occurrence and development of colorectal cancer and become a potential treatment method (107, 108). Recent research suggests that CTLA4 in combination with some shRNAs (sh-circQSOX1) can effectively reduce the resistance of colorectal cancer immunotherapy (109). Additionally, targeting circRNA with antisense oligonucleotides (ASOs) can reduce the invasion and metastasis of CRC (110). Innovative treatment strategies include using engineered exogenous circRNAs (cloning circRNA into a virus and transfecting CRC cells) as molecular sponges for oncogenic miRNAs to inhibit tumor growth (111) and developing new circRNA vaccines (such as recently discovered SARS-CoV-2 circRNA vaccines) (112).

## 10 POLE/POLD1 mutation

POLE and POLD1 are genes that encode the catalytic subunits of DNA polymerase ϵ and DNA polymerase δ, respectively. Pathogenic variants (PVs) within their exonuclease domain (ED) can lead to loss of cell proofreading function and the generation of numerous neoantigens, resulting in a better response to immunotherapy. However, not all mutations located within the exonuclease domain are meaningful. Currently, common and pathogenic hotspot mutations include P286R, V411L, S297F, A456P, and S459F. In recent years, researchers have discovered some mutations that are pathogenic despite not being located within the exonuclease domain, such as POLE V1368M (113).

### 10.1 The correlation with pathological features

Earlier studies analyzing 6517 CRC patients found that POLE somatic mutations are more common in males, right-sided colon cancer, and early-stage patients, and are associated with a favorable prognosis (114). However, in recent years, Hu et al. discovered for the first time that different regions may significantly influence the primary sites of POLE-driven mutations: in the Asian population, POLE-driven mutations are more likely to occur in the left colon (left vs. right: 77.78% vs. 11.11%), while non-Asian patients are more likely to occur in the right colon (115).

### 10.2 The relationship with MSI

Similar to MSI-H tumors, tumors with POLE/POLD1 mutations generally exhibit high tumor mutation burden (TMB). However, the mutations in the latter generally exceed 100 mut/Mb, also known as hypermutation. Moreover, most colorectal cancers with POLE/POLD1 mutations exhibit a microsatellite stable (MSS) phenotype. Compared to patients with POLE wild-type or non-exonuclease domain mutations (POLE non-EDMs), patients with POLE EDMs have a higher frequency of MSI-H (115). However, there is still some controversy regarding the sequence of occurrence of MSI-H and POLE/POLD1 mutations in tumors. The current mainstream view seems to favor POLE mutations as the driving factor of dMMR/MSI-H (116). However, some scholars hold the opposite view, suggesting that dMMR can significantly increase TMB in tumors by affecting POLE function (117). More research is still needed to confirm the exact order of occurrence of the two.

### 10.3 Predictive value and treatment strategies

POLE/POLD1 has been recognized as a biomarker for CRC therapy. In a retrospective study, patients with pathogenic POLE/POLD1 mutations who received PD-1/PD-L1 monotherapy or combination therapy with CTLA-4 inhibitors showed significantly higher clinical benefit rates and improved survival outcomes compared to patients with benign mutations (113). Another study involving over 2500 patients found that 75% of tumors with pathogenic POLE/POLD1 mutations responded well to ICI therapy, either achieving remission or showing significant improvement in prognosis post-immunotherapy Functional landscapes of pole and pold1 mutations in checkpoint blockade-dependent antitumor immunity. In a survival analysis of stage II CRC patients, those carrying POLE ED mutations had excellent prognosis regardless of MSI status. Thus, POLE/POLD1 mutations can be considered as a biomarker independent of MSI-H/dMMR (118).

Considering the rarity of this mutation, there are still relatively few prospective clinical studies ongoing. Currently, a phase II clinical trial (NCT03810339) is underway, investigating the use of toripalimab in the treatment of advanced solid tumors with POLE/POLD1 mutations, with the hope of reaching conclusions soon.

## 11 RET fusions

RET is an oncogene that encodes a transmembrane receptor with a tyrosine kinase domain. Recent findings have linked RET to intestinal motility function, with its signaling persisting in the adult intestine. It can stimulate intestinal motility by limiting the release of PYY from enteroendocrine cells, and this mechanism primarily occurs in adult males (119, 120). Activation of RET can initiate downstream signaling pathways such as RAS/MAPK, PI3K/AKT, JAK–STAT, or JNK, leading to excessive cell proliferation and promoting tumorigenesis. RET activation mechanisms include mutations and fusions, each with distinct clinical and pathological features. Here, we focus on the clinical significance of RET fusions in CRC.

RET fusions are not common in colorectal cancer, accounting for less than 1%, but research on this mechanism is relatively abundant. In colorectal cancer, NCOA4-RET is the most common fusion variant, accounting for approximately 46%. RET fusion is associated with older age, right-sided colon location, RAS/BRAF wild-type, MSI-H, and worse prognosis, while also exhibiting a higher median TMB. Therefore, it can be identified as a distinct molecular subgroup of colorectal cancer (121). The 2024 V1 version of the colorectal cancer NCCN guidelines includes RET fusion genes as recommended biomarkers for testing. Therefore, testing for RET fusion genes in these “advantaged populations” can help determine the prognosis of mCRC patients and seek treatment opportunities. A multicenter, phase 1/2, basket study published in Nature Medicine included 45 patients with RET gene fusions (including 10 patients with colorectal cancer) and found significant anti-tumor activity of Selpercatinib in patients with RET fusion-positive advanced colorectal cancer. In addition to RET fusion (especially NCOA4), TMB, and TP53 mutation status may influence the efficacy of selpercatinib (122).

## 12 Conclusion

Currently, KRAS, BRAF, and MSI status play a crucial role in predicting resistance in CRC patients. Therefore, routine testing for these genes is necessary in CRC patients to determine subsequent treatment plans. Among these, MSI status has the highest relevance, as even advanced MSI CRC patients have a high chance of long-term survival after immunotherapy (29). Research on HER-2 in colorectal cancer is becoming increasingly profound, and its strong predictive ability is recognized. However, few drugs targeting HER-2 have been approved for use in colorectal cancer, making the development of anti-HER-2 drugs a current priority (123).

In recent years, new biomarkers have emerged, such as inflammation-related indicators like the lymphocyte-to-monocyte ratio (LMR), which not only predicts overall survival in colorectal cancer patients but also shows better predictive value than some conventional biomarkers like neutrophil-to-lymphocyte ratio (NLR), platelet-to-lymphocyte ratio (PLR), and modified Glasgow Prognostic Score (mGPS) (124). Intestinal microbiota such as Fusobacterium nucleatum has also been identified as a predictive biomarker for colorectal cancer (125). The emergence of consensus molecular subtypes can further divide CRC patients into different subgroups, providing better guidance for patient treatment and enabling more precise personalized treatment by doctors.

Furthermore, new biomarker detection methods are rapidly evolving, such as liquid biopsy, a minimally invasive method that can detect components of cancer tissue origin in the blood, allowing real-time monitoring of tumor dynamics. The detection of ctDNA and non-coding RNA may play important roles in predicting recurrence, monitoring metastasis, and guiding treatment. However, due to issues such as low molecular content from tumor sources and low mutation signal intensity, as well as the frequency of monitoring still under debate, their clinical application is not yet widespread. Recently, new research results similar to ctDNA were published in NEJM: blood cfDNA (sensitivity for colorectal cancer was 83%, for precancerous lesions was 13%) and a second-generation multi-target fecal DNA detection method (sensitivity for colorectal cancer was 93.9%, for precancerous lesions was 43.4%). The achievements of these two major studies signify significant progress in colorectal cancer detection technology and methods (126, 127). Other emerging biomarkers such as POLE/POLD1 and RET, though still in early validation phases, have expanded stratification and therapeutic approaches for colorectal cancer patients. Therefore, it may currently be necessary to integrate multiple biomarkers to design a novel predictive model that enhances and refines risk stratification in colorectal cancer and guides personalized treatment strategies. However, the critical challenge lies in integrating these biomarkers into clinical decision-making frameworks. Current evidence supporting their clinical utility remains limited, and more multicenter studies are required to assess the feasibility and safety of clinical translation for these biomarkers.

Considering that we are in the era of personalized medicine, focusing on biomarker detection and development, gaining a deeper understanding of potential mechanisms of treatment resistance, and developing new treatment targets are the major trends in future colorectal cancer research.
