# Correction: Prognostic and predictive molecular biomarkers in colorectal cancer

**DOI:** 10.3389/fonc.2025.1725836

**Published:** 2025-10-27

**Authors:** Jianzhi Zhang, Hao Zhu, Wentao Liu, Ji Miao, Yonghuan Mao, Qiang Li

**Affiliations:** ^1^ Department of General Surgery, Nanjing Drum Tower Hospital, Affiliated Hospital of Medical School, Nanjing University, Nanjing, China; ^2^ Department of General Surgery, Nanjing Drum Tower Hospital Clinical College of Nanjing Medical University, Nanjing, China; ^3^ Department of General Surgery, Affiliated Drum Tower Hospital, Nanjing University of Chinese Medicine, Nanjing, China; ^4^ Department of General Surgery, Affiliated Drum Tower Hospital, JiangSu University, Nanjing, China

**Keywords:** colorectal cancer, biomarkers, circulating tumor DNA, non-coding RNA, POLE/POLD1, RET

Affiliation “Department of General Surgery, Nanjing Drum Tower Hospital, Affiliated Hospital of Medical School, Nanjing University, Nanjing, China” was erroneously given as “Department of General Surgery, Affiliated Drum Tower Hospital, Nanjing University Medical School, Nanjing, China”.

The original version of this article has been updated.

